# *Crataegus pinnatifida* Bunge Inhibits RANKL-Induced Osteoclast Differentiation in RAW 264.7 Cells and Prevents Bone Loss in an Ovariectomized Rat Model

**DOI:** 10.1155/2021/5521562

**Published:** 2021-03-27

**Authors:** Minsun Kim, MinBeom Kim, Jae-Hyun Kim, SooYeon Hong, Dong Hee Kim, Sangwoo Kim, Eun-Young Kim, Hyuk-Sang Jung, Youngjoo Sohn

**Affiliations:** Department of Anatomy, College of Korean Medicine, Kyung Hee University, Seoul 02447, Republic of Korea

## Abstract

Osteoporosis is characterized by a decrease in bone microarchitecture with an increased risk of fracture. Long-term use of primary treatments, such as bisphosphonates and selective estrogen receptor modulators, results in various side effects. Therefore, it is necessary to develop alternative therapeutics derived from natural products. *Crataegus pinnatifida* Bunge (CPB) is a dried fruit used to treat diet-induced indigestion, loss of appetite, and diarrhea. However, research into the effects of CPB on osteoclast differentiation and osteoporosis is still limited. *In vitro* experiments were conducted to examine the effects of CPB on RANKL-induced osteoclast differentiation in RAW 264.7 cells. Moreover, we investigated the effects of CPB on bone loss in the femoral head in an ovariectomized rat model using microcomputed tomography. *In vitro*, tartrate-resistant acid phosphatase (TRAP) staining results showed the number of TRAP-positive cells, and TRAP activity significantly decreased following CPB treatment. CPB also significantly decreased pit formation. Furthermore, CPB inhibited osteoclast differentiation by suppressing NFATc1, and c-Fos expression. Moreover, CPB treatment inhibited osteoclast-related genes, such as *Nfatc1, Ca2, Acp5, mmp9, CtsK, Oscar*, and *Atp6v0d2*. *In vivo*, bone mineral density and structure model index were improved by administration of CPB. In conclusion, CPB prevented osteoclast differentiation *in vitro* and prevented bone loss *in vivo.* Therefore, CPB could be a potential alternative medicine for bone diseases, such as osteoporosis.

## 1. Introduction

Osteoporosis is characterized by a decrease in bone microarchitecture and an increased risk of fracture [1]. Bone remodeling is balanced between bone formation by osteoblasts and bone resorption by osteoclasts [2]. However, the excessive activity of osteoclasts induces osteoporosis, rheumatoid arthritis, and periodontitis. Thus, the inhibition of the osteoclast differentiation and its activity plays a role in the treatment strategy of osteoporosis.

Osteoclasts are *giant*, multinucleated cells derived from hematopoietic stem cells. Receptor activator of nuclear factor kappa-*β* ligand (RANKL) is essential for osteoclast differentiation [3, 4]. The binding of RANKL to RANK stimulates tumor necrosis factor receptor (TNFR)-associated factor 6 (TRAF6), activating mitogen-activated protein kinase (MAPKs) and nuclear factor kappa-*β* (NF-*κ*B). As a result, it induces the expression of NFATc1 and c-Fos, known as essential transcription factors for osteoclast differentiation. These transcription factors induce the expression of osteoclast-related genes such as tartrate-resistant acid phosphatase (TRAP), carbonic anhydrase II (CA2), matrix metallopeptidase 9 (MMP-9), ATPase H+ transporting lysosomal 38 kDa V0 subunit d2 (ATP6v0d2), osteoclast associated receptor (OSCAR), and cathepsin K (CTK) [5, 6].

Bisphosphonate and selective estrogen receptor modulators (SERMs) are frequently used as treatments. However, long-term treatment of these agents causes side effects such as Paget's disease of bone, breast cancer, prostate cancer, hot flashes, and night sweats [7–10]. Therefore, there is a need for integrating complementary and alternative medicines for osteoporosis based on natural products with few side effects. Consequently, the importance of developing an alternative treatment for osteoporosis has increased currently.

*Crataegus pinnatifida* Bunge (CPB) is the dried fruit of *Crataegus pinnatifida* Bung, called “Sansa” in Korea [11]. Previous studies have shown that CPB has antioxidant and anti-inflammatory effects [12, 13]. Chlorogenic acid is the major component of *Crataegus pinnatifida* Bunge and has an inhibitory effect on osteoclast differentiation induced by RANKL [14]. It has also been linked to anti-inflammatory and antioxidant effects [15, 16]. Osteoporosis is caused by endocrine, metabolic, and mechanical factors. Furthermore, recent studies have shown that the risk of developing osteoporosis is increased in inflammatory conditions [17, 18]. Therefore, we hypothesize that CPB may have a positive effect on bone metabolism.

In this study, we investigated the *in vitro* effects of CPB on RANKL-induced osteoclast differentiation. In addition, we also investigated the *in vivo* effects of CPB on bone loss in an ovariectomized (OVX) model.

## 2. Materials and Methods

### 2.1. Chemicals and Reagents

RANKL was purchased from PeproTech (London, UK). Dulbecco's modified eagle medium (DMEM) was purchased from Welgene (Daejeon, Korea). Minimum Essential Medium Eagle alpha-modification (*α*-MEM), penicillin/streptomycin (P/S), Dulbecco's phosphate buffered saline (DPBS), and normal serum were purchased from Gibco (Gaithersburg, MD). Fetal bovine serum (FBS) was supplied by Atlas Biologicals (Fort Collins, CO). TRAP staining kit, bicinchoninic acid (BCA) solution, and 17*β*-estradiol (E_2_) were obtained from Sigma Aldrich (St. Louis, MI, USA). Cell Titer 96® AQueous nonradioactive cell proliferation assay (MTS) was obtained from Promega (Madison, WI, USA). Osteo assay surface multiple well plate was obtained from Corning Inc. (Corning, NY, USA). Anti-*β*-actin (cat. no. sc-8432) and anti-c-Fos (cat. no. sc-447) were supplied Santa Cruz Biotechnology (Santa Cruz, CA, USA). Anti-NFATc1 (cat. no. 556602) was supplied by BD Pharmingen (San Diego, CA, USA). Secondary antibodies (cat. no. 111-035-045, 115-035-062) were supplied by Jackson ImmunoResearch (West Grove, PA, USA). Polymerase chain reaction (PCR) primers were obtained from Genotech (Daejeon, Korea). SuperScript II Reverse transcription kit and SYBR green were obtained from Invitrogen (Carlsbad, CA, USA). Taq polymerase was obtained from Kapa Biosystems (Woburn, MA, USA). Avidin-biotin complex (ABC) kit and 3,3′-diaminobenzidine (DAB) were obtained from Vector Laboratories, Inc. (Burlingame, CA, USA). All reagents used in the experiments were of analytical grade.

### 2.2. Preparation of CPB Extract

CPB was obtained from Omni Herb Inc. (Seoul, Korea). The sample extract was prepared by decocting 600 g dried herb with 6 L boiling distilled water (dH_2_O) for 2 h. Next, the filtrate was evaporated using a vacuum evaporator and freeze-dried into powder. The yield from the dried herbs was 39.6% (freeze-dried powder: 237.8 g), and the powder was subsequently stored at −20°C.

### 2.3. High-Performance Liquid Chromatography Analysis

Quantitative analysis of main components in CPB was performed using an A Waters 2695 system equipped with a Waters 2487 Dual *λ* absorbance detector and X-bridge C18 Column (250 mm × 4.6 mm, 5 *μ*m). CPB dissolved in dH_2_O. CPB was passed through a 0.2-*μ*m membrane filter and 10 *μ*L volume of the filtrate was injected into the HPLC column. The mobile phases are composed of solvent A (acetonitrile) and solvent B (H_2_O (1% acetic acid)). The detection time was 0–30 min. The flow rate was 1.0 mL/min.

### 2.4. Cell Culture and Cell Viability

The RAW 264.7 cells were purchased from Korean Cell Line Bank (Seoul, Korea). RAW 264.7 cells were cultured in DMEM supplemented with 1% P/S and 10% FBS. The cells were incubated at 37°C in a humidified atmosphere of 5% CO_2_ (Thermo Fisher, Waltham, MA, USA). The MTS assay was performed to examine the toxicity of CPB on RAW 264.7 cells. RAW 264.7 cells were seeded at a density of 5 × 10^3^ cells/well in a 96-well plate. The CPB was administered at 125, 250, 500, and 1000 *μ*g/mL for 24 h. Afterwards, 20 *μ*L MTS solution was added to the wells for 2 h. The absorbance (490 nm) was measured by an enzyme-linked immunosorbent assay (ELISA) reader. Results were indicated as a percentage of the control. Cytotoxicity was considered as cell viability less than 90% of the control.

### 2.5. TRAP Staining and Pit Assay

RAW 264.7 cells were seeded at a density of 5 × 10^3^ cells/well in 96-well plate. After 24 h, RAW 264.7 cells were differentiated with *α*-MEM supplemented with 1% P/S, 10% FBS, and RANKL (100 ng/mL). The media was changed every 2 days. After 5 days, the osteoclast cells were fixed with 10% formalin for 10 min and then stained using a TRAP staining kit, according to the manufacturer's instructions. Afterwards, cells were rinsed with dH_2_O and dried at room temperature. Multinucleated osteoclasts were considered as TRAP-positive cells with three or more nuclei (red color). To measure TRAP activity, differentiation medium was transferred to new 96-well plates and TRAP solution (4.93 mg pNPP + 850 *μ*L 0.5 M acetate solution + 150 *μ*L tartrate solution) was added to 96-well plate at 37°C for 1 h. TRAP activity was measured at 405 nm by an ELISA reader. To examine pit formation, RAW 264.7 cells were seeded at a density of 5 × 10^3^ cell/well in a multiple well osteo assay surface plate and incubated for 5 days. Thereafter, the cells were removed using NaClO. The pit area was measured by ImageJ version 1.46 (National Institutes of Health, Bethesda, MD, USA).

### 2.6. Western Blotting

RAW 264.7 cells were incubated with RANKL and various concentrations of CPB extract (125, 250, 500, and 1000 *μ*g/mL) for 24 h. Cells were lysed in radio-immunoprecipitation assay (RIPA) lysis buffer (50 mM Tris-Cl, 150 mM NaCl, 1% NP–40, 0.5% sodium deoxycholate, and 0.1% SDS) consisting of proteinase inhibitors (Sigma Aldrich; Merck KGaA, Darmstadt, Germany) to obtain the protein. Thereafter, total protein quantification was done using BCA assay according to the manufacturer's instructions. The protein samples were separated by 10% sodium dodecyl sulphate-polyacrylamide gel electrophoresis (SDS-PAGE) and transferred onto a nitrocellulose membrane. The membranes were blocked (5% skim milk) for 1 h and incubated overnight at 4°C with primary antibodies for *β*-actin (1 : 1,000), NFATc1 (1 : 1,000), and c-Fos (1 : 1,000). After 24 h, the membranes were incubated with secondary antibodies (1 : 10,000) for 1 h at room temperature. The protein was visualized by enhanced chemiluminescence (ECL) (Whatman plc; GE Healthcare) and protein band densitometry was measured by ImageJ version 1.46. All data were normalized to the *β*-actin density.

### 2.7. Reverse Transcription-Quantitative PCR (RT-qPCR)

RAW 264.7 cells were incubated with CPB (125, 250, 500, and 1000 *μ*g/mL) for 4 days and the RANKL (100 ng/mL). Total RNA was extracted from RAW 264.7 cells using TRIzol reagent, according to the manufacturer's instructions. Then, cDNA was synthesized using the reverse transcription kit (Invitrogen, Carlsbad, CA, USA). RT-qPCR was performed with a C1000 Touch™ thermal cycler (Bio-Rad Laboratories, Hercules, CA, USA) and Taq polymerase. The PCR cycling conditions were initial denaturation cycle at 95°C for 5 min, followed by 30–40 cycles of amplification at 94°C for 30 sec, annealing at 53–58°C for 30 sec, and extension at 72°C for 30 sec. Primers for osteoclast-related genes are described in Table 1. The reaction was electrophoresed on 1–1.2% agarose gels stained with SYBR. The agarose gel was visualized using N*α*B^I^™ (Neoscience, Suwon, Korea). The expression level of mRNA in the analyzed gene was normalized to the glyceraldehyde-3-phosphate dehydrogenase (GAPDH) using ImageJ version 1.46.

### 2.8. Animal Experiments and Induction of OVX Rats

Animal experiments were conducted in accordance with Guidelines for the Care and Use of Laboratory Animals approved by the Committee on Animal Experimentation of Kyung Hee University (KHUASP (SE)-15-101). Female Sprague–Dawley (SD) rats (12 weeks of age) were purchased from Nara Biotech (Seoul, Korea). SD-rats were housed at 22 ± 2°C, with a relative humidity of 53–55% in a 12 h light-dark cycle. In this study, all animals had *ad libitum* access to water and food. The SD-rats were acclimatized for one week before surgery. To establish an OVX model, the rats were anesthetized with 100% oxygen and 5% isoflurane to remove the ovaries. The sham group did not have their ovaries removed but received the same stress. The rats were divided into five groups (*n* = 8 per group) as follows: (1) sham group; sham-operation, treated with dH_2_O, (2) OVX group; OVX-induced, treated with dH_2_O, (3) E_2_ group; OVX-induced, treated with 100 *μ*g/kg 17*β*-estradiol, (4) CPB-L group; OVX-induced, treated with 13.2 mg/kg CPB, and (5) CPB-H group; OVX-induced, treated with 132 mg/kg CPB. The dose of CPB was calculated as follows: In Korean medicine, the recommended single dose for an adult is 8 g/60 kg, effectively equating to 3.168 g (yield, 39.6%) CPB powder of 8 g dried herbs. Therefore, the CPB-L group was administered 13.2 mg/kg CPB. Since the metabolism of rodents is faster than that of humans, the high-dose group was administered 10 times the concentration of the low-dose group [19]. Thus, CPB-H group was administered 132 mg/kg CPB. To prevent infection at the surgical site, all rats received injections with 4 mg/kg gentamicin for 3 days after surgery. E_2_ and CPB were dissolved in dH_2_O and administered orally once per day for 8 weeks. Body weights were measured once a week. During the experiments, all animals showed no side effects and exhibited no abnormal behavior. After 8 weeks, the experimental animals were anesthetized with 100% oxygen and 5% isoflurane and sacrificed by lethal cardiac puncture and cervical vertebrae dislocation.

### 2.9. Microcomputed Tomography Analysis

After sacrifice, the femur samples were fixed in 10% neutral buffered formalin (NBF) at room temperature for 24 h. The femoral head was analyzed using microcomputed tomography (micro-CT) (SkyScan1176; Bruker Corporation, Kontich, Belgium). Bone microstructure parameters such as bone mineral density (BMD), bone volume/total volume (BV/TV), and structure model index (SMI) were analyzed using NRecon software (SkyScan version 1.6.10.1; Bruker Corporation, Billerica, MA, USA).

### 2.10. Hematoxylin and Eosin (H&E) Staining

The fixed femur samples were decalcified in ethylenediaminetetraacetic acid (EDTA) for 4 weeks at room temperature. Afterwards, femur samples were dehydrated and embedded in paraffin. Femur samples were sectioned using a rotary microtome (5 *μ*m-thick, ZEISS, Oberkochen, Germany), then dried and stained with H&E. Changes in tissue parameters, such as femoral head area, were observed using an inverted light microscope (magnification, 40x and 100x; Olympus Corporation, Tokyo, Japan). The trabecular area was measured by ImageJ version 1.46.

### 2.11. Immunohistochemistry Staining

Sectioned tissues were paraffinized and rehydrated to prepare for immunohistochemistry (IHC). Femur tissue slides were treated with 0.3% hydrogen peroxide-methanol to inhibit endogenous peroxidase. Subsequently, nonspecific reactions were blocked with normal serum for 1 h at room temperature. After washing thrice with PBS, sections were incubated with primary antibody at 4°C overnight and then incubated with secondary antibodies for 1 h at room temperature. The tissues were incubated with an ABC kit for 30 min at room temperature, followed by staining with DAB solution and counterstaining with hematoxylin. Histological changes were analyzed using a light microscope (magnification, 100x and 200x).

### 2.12. Statistical Analysis

Data are presented as mean ± standard error (SEM) of the mean for three replicates. Differences between the control and CPB treatment groups were analyzed using one-way ANOVA followed by a Dunnett's post hoc in GraphPad PRISM version 5.01 (GraphPad Software Inc., San Diego, CA, USA). Statistical significance was determined at *p* < 0.05.

## 3. Results

### 3.1. Quantitative Analysis of the CPB Extract

**Figure 1:**
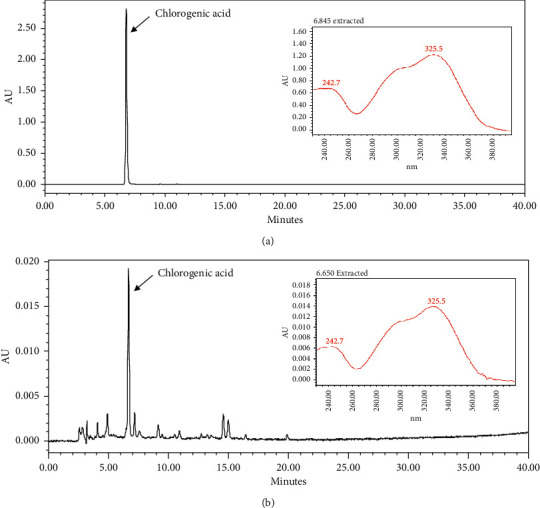
Quantitative HPLC of (a) chlorogenic acid standard and (b) CPB. The HPLC-analysis for standards and sample solutions. (a) Chlorogenic acid standard solution; (b) CPB samples were detected at 330 nm.

HPLC was used to confirm the main component of CPB [20]. As shown in Figure 1, the retention times of CPB are identical to the retention times of the chlorogenic acid standards.

### 3.2. Effect of CPB on RANKL-Induced TRAP Activity and Pit Formation

**Figure 2:**
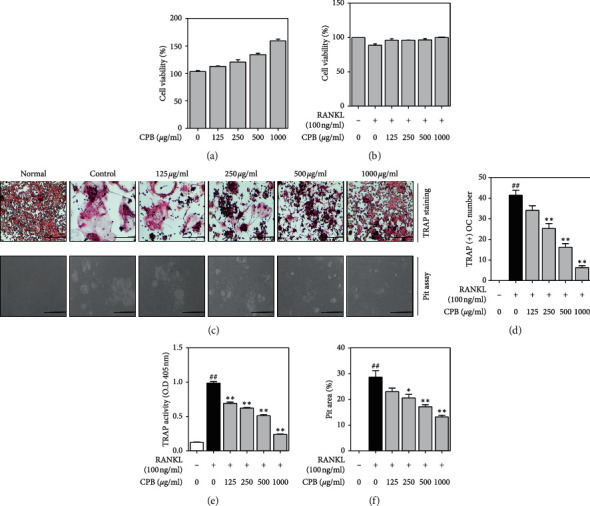
Effect of CPB on cell viability, osteoclast differentiation, and bone formation. (a) RAW 264.7 cells were measured by MTS assay of CPB treatment for 24 h. (b) After differentiation into osteoclasts for 5 days, cytotoxicity was measured using MTS. (c) TRAP-positive cells and pit area were captured using an inverted microscope (100x, Scale bars: 200 *μ*m). (d) TRAP-positive cells were counted with an inverted microscope. (e) TRAP activity was measured with an ELISA reader (405 nm). (f) The pit area was measured with ImageJ version 1.46 (100x, Scale bars: 200 *μ*m). The results are presented as the mean ± SEM (*n* = 3). ^##^*p* < 0.01 compared to the normal group (untreated cells), and ^*∗∗*^*p* < 0.01, ^*∗*^*p* < 0.05 compared to the control group (only-RANKL treated cells).

To determine the cytotoxic effect of CPB, RAW 264.7 cells and osteoclast were treated with CPB concentrations from 125, 250, 500, and 1000 *μ*g/mL. In this study, none of the CPB concentrations affected cell viability in either RAW 264.7 cells or osteoclasts (Figures 2(a) and 2(b)). To investigate the effect of CPB on RANKL-induced osteoclast differentiation and pit formation, TRAP staining and pit assay was used. RANKL increased the number of TRAP-positive cells and TRAP activity compared with the untreated control group, confirming osteoclast differentiation. CPB treatment of differentiated osteoclasts decreased the number of TRAP-positive cells and TRAP activity in a dose-dependent manner. In addition, the pit area was increased with RANKL treatment, compared to untreated controls, and decreased by CPB treatment in a dose-dependent manner (Figures 2(c)–2(f)).

### 3.3. Effect of CPB on RANKL-Induced Expression of NFATc1 and c-Fos

**Figure 3:**
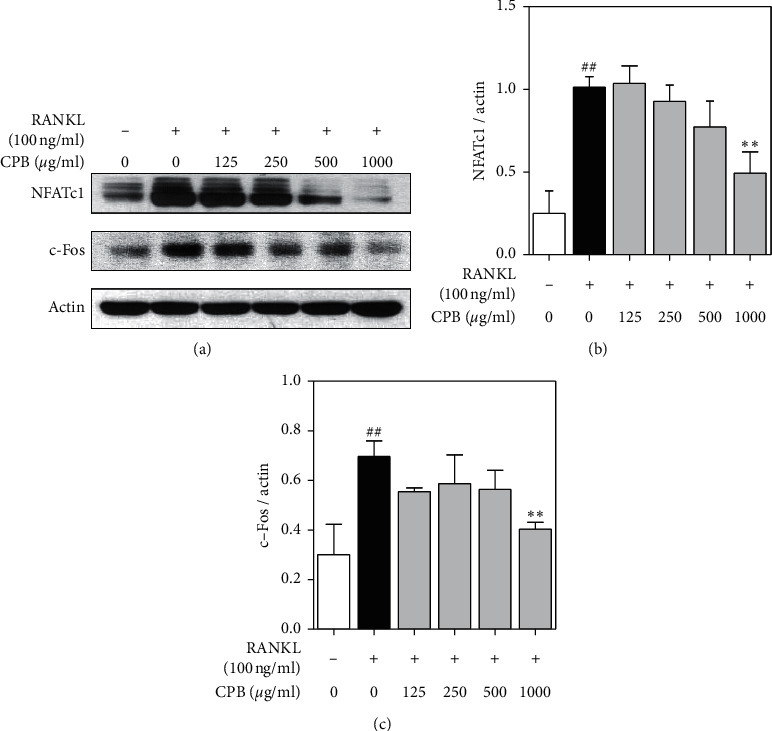
Effect of CPB extract on transcription factor such as NFATc1 and c-Fos. (a) RAW 264.7 cells were treated with RANKL (100 ng/mL) and CPB treatment for 24 h. The expressions of NFATc1 and c-Fos were determined by western blotting. (b) NFATc1 and c-Fos were normalized to Actin with ImageJ version 1.46. The results are presented as the mean ± SEM (*n* = 3). ^##^*p* < 0.01 compared to the normal group (untreated cells), and ^*∗∗*^*p* < 0.01, ^*∗*^*p* < 0.05 compared to the control group (only-RANKL treated cells).

To examine the expression of NFATc1 and c-Fos, we performed western blotting (Figures 3(a) and 3(b)). NFATc1 and c-Fos expression were significantly increased in the RANKL-induced cells compared to the nonstimulated control group. Therefore, the expressions of NFATc1 and c-Fos were suppressed by CPB in a dose-dependent manner.

### 3.4. Effect of CPB on RANKL-Induced of Osteoclast-Related Genes

**Figure 4:**
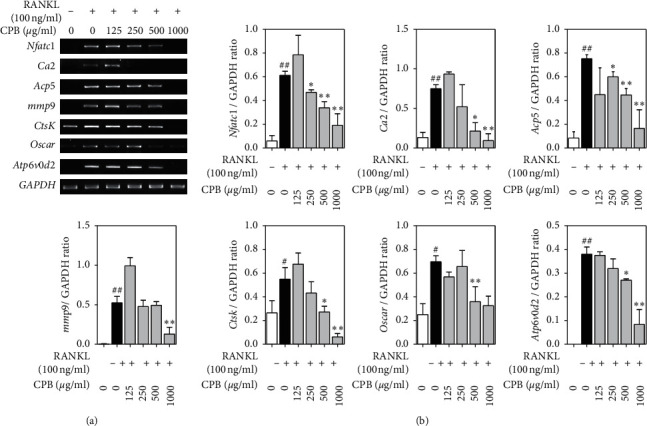
Effect of CPB extract on osteoclast-related genes. (a) RAW 264.7 cells were treated with RANKL (100 ng/mL) and CPB treatment for 4 days. RT-qPCR was used to determine the mRNA levels of osteoclast-related genes. (b) The levels of mRNA were normalized to GAPDH. The results are presented as the mean ± SEM (*n* = 3). ^##^*p* < 0.01, ^#^*p* < 0.05 compared to the normal group (untreated cells), and ^*∗∗*^*p* < 0.01, ^*∗*^*p* < 0.05 compared to the control group (only-RANKL treated cells).

To investigate the effect of CPB on osteoclast-related genes in RANKL-induced RAW 264.7 cells, RT-qPCR was performed. Treatment with RANKL increased the mRNA levels of *Nfatc1*, *Ca2*, *Acp5*, *mmp9*, *CtsK*, *Oscar*, and *Atp6v0d2*. In contrast, CPB reduced these mRNA levels in a dose-dependent manner, the most effective dose in all instances (Figures 4(a) and 4(b)).

### 3.5. Effect of CPB on OVX-Induced Models

**Figure 5:**
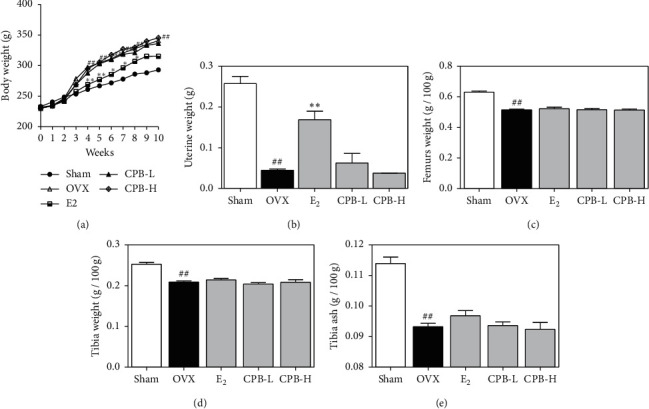
Effect of CPB on OVX-induced model. (a) The body weight was measured once a week. (b) Uterus weight, (c) femurs weight, (d) tibia weight, and (e) tibia ash was measured after sacrifice. The results are presented as the mean ± SEM of each experimental group (*n* = 8). ^##^*p* < 0.01, ^#^*p* < 0.05 compared to the normal group (sham group), and ^*∗∗*^*p* < 0.01, ^*∗*^*p* < 0.05 compared to the control group (OVX group).

To analyze the effect of CPB on OVX-induced postmenstrual osteoporosis, we orally administered E_2_, CPB-L, and CPB-H to the OVX-induced rats daily for 8 weeks. As shown in Figure 5(a), the body weight of both treated and untreated OVX-induced rats significantly increased after 3 weeks as compared to that of the sham group. However, there was no significant difference in body weight between the OVX group and E_2_, CPB-L, and CPB-H, respectively. The uterus weight decreased in the OVX group as compared with that in the sham group (Figure 5(b)). Furthermore, the uterus weight increased in the E_2_ group as compared with that in the OVX group, with no effect observed in CPB-L and CPB-H, femur weights significantly decreased in the OVX group compared to that in the sham group (Figure 5(c)). However, there was no difference in femur weight between the E_2_, CPB-L, and CPB-H groups compared to the OVX group. Tibia weight and ash were decreased in the OVX group compared with the sham group (Figures 5(d) and 5(e)), though there were no significant differences between E_2_, CPB-L, and CPB-H, compared to the OVX group.

### 3.6. Effect of CPB on Bone Loss in OVX-Induced Models

**Figure 6:**
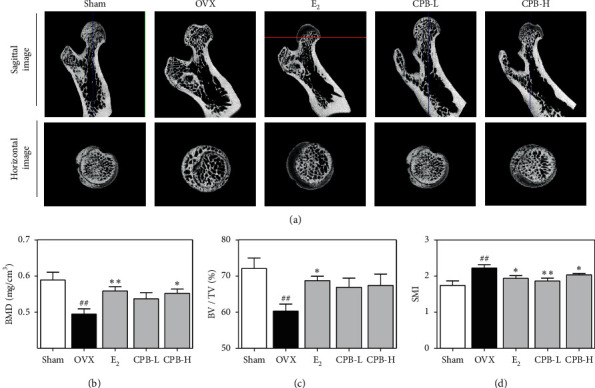
Effect of CPB on an osteoporosis rat model. (a) Analysis of micro-CT in the femoral head. The bone microstructure parameters, such as (b) BMD, (c) BV/TV, and (d) SMI, were measured by micro-CT. The results are presented as the mean ± SEM for each experimental group (*n* = 8). ^##^*p* < 0.01, ^#^*p* < 0.05 compared to the normal group (sham group), and ^*∗∗*^*p* < 0.01, ^*∗*^*p* < 0.05 compared to the control group (OVX group).

In the micro-CT image, the bone density in the femoral head of OVX group was decreased compared with the sham group (Figure 6(a)). Furthermore, E_2_ and CPB-H significantly increased bone density in the femoral head compared with the OVX group. From the results of the bone microstructure analysis, BMD was significantly decreased in the OVX group compared to sham (Figure 6(b)), while in E_2_ and CPB-H groups, BMD increased compared with the OVX group. As shown in Figure 6(c), BV/TV was decreased in the OVX group compared with the sham group. E_2_ significantly increased BV/TV compared to the OVX group. CPB-L and CPB-H groups had increased BV/TV but not significantly. In addition, SMI was increased in the OVX group compared to the sham group (Figure 6(d)). In contrast, SMI was reduced in all three groups: E_2_, CPB-L, and CPB-H, compared to the OVX group.

### 3.7. Effect of CPB on Trabecular Area and Expression of CTK in the Femoral Head

**Figure 7:**
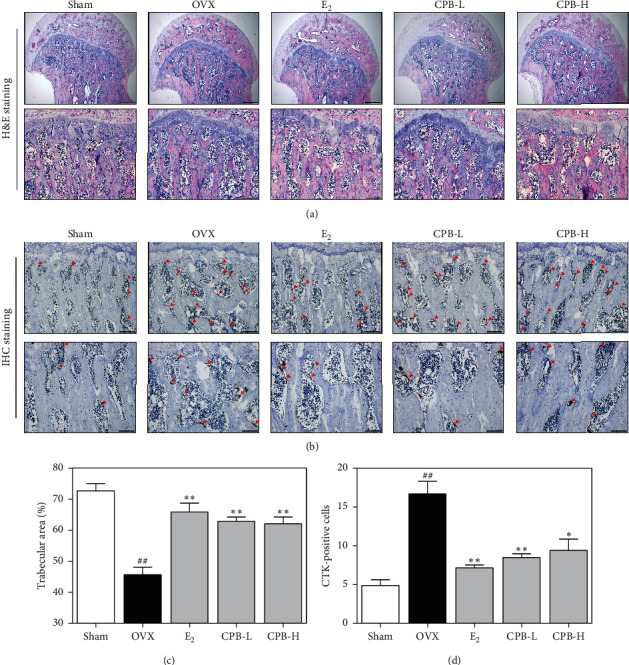
Effect of CPB on OVX-induced bone loss model. (a) The histology of the bone tissues was examined using H&E staining, and (b) IHC staining. (c) The trabecular area was measured using ImageJ version 1.46. (d) CTK-positive cells were counted using ImageJ version 1.46. CTK-positive cells are indicated by red arrows. The results are presented as the mean ± SEM for each experimental group (*n* = 8). ^##^*p* < 0.01, ^#^*p* < 0.05 compared to the normal group (sham group), and ^*∗∗*^*p* < 0.01, ^*∗*^*p* < 0.05 compared to the control group (OVX group).

To measure the trabecular area, bone tissues were stained with H&E (Figure 7(a)). To determine the effect of CPB treatments on the CTK in OVX-induced rats, we perform the IHC staining (Figure 7(b)). The trabecular area was decreased in the OVX group when compared with that of the sham group. Treatments with E_2_, CPB-L, and CPB-H inhibited the loss of the trabecular area compared with that of the OVX group (Figure 7(c)). Furthermore, OVX groups significantly increased CTK compared to the sham group. Concurrently, E_2_, CPB-L, and CPB-H groups reduced CTK compared to the OVX group (Figure 7(d)).

## 4. Discussion

According to recent studies, various side effects have been reported with the administration of bisphosphonate and SERM, which are currently used for the treatment of osteoporosis. This has prompted many researchers to search for safer alternative medicinal agents with fewer side effects for osteoporosis treatment [7–10]. In this study, we examined the osteoclastogenesis and antiosteoporosis effects of CPB on RAW 264.7 cells. *In vitro*, CPB demonstrated an inhibitory effect on osteoclast differentiation by inhibiting transcription factors and osteoclast-related genes*. In vivo*, CPB also prevented bone loss in OVX-induced rat models.

TRAP is a known osteoclast phenotype marker, and TRAP staining is a standard method used to determine osteoclast expression and activation [21, 22]. In the present study, TRAP staining results showed a significant decrease in TRAP-positive cells and TRAP activity following CPB treatment. Pit formation is commonly used to measure the osteoclasts' differentiation and bone resorption ability [23, 24]. As a result of the experiment, CPB significantly suppressed the pit area. It is unclear whether CPB reduces pit formation by inhibiting the ability of osteoclasts to bone resorption, or it controls pit formation by inhibiting osteoclast differentiation, but the TRAP staining and pit assay results, CPB, seem to regulate bone resorption by inhibiting osteoclast differentiation.

Transcription factors, such as NFATc1 and c-Fos, are essential in osteoclast differentiation [25, 26]. In a previous study, c-Fos-deficient cells were not able to differentiate into osteoclasts [11]. In contrast, excessive expression of c-Fos causes osteosarcoma and chondrosarcoma [27]. Furthermore, NFATc1-deficient mice develop osteopetrosis due to blocked osteoclast differentiation [28]. It has also been reported that embryonic stem cells deficient in NFATc1 cannot differentiate into osteoclasts upon RANKL stimulation [29]. Therefore, c-Fos and NFATc1 are important factors for osteoclast differentiation [25, 26]. The present study showed that CPB significantly decreased the expression of c-Fos and NFATc1 and subsequent osteoclast differentiation.

c-Fos regulates bone resorption markers, such as CA2, which acidifies the bone surface during bone resorption [30–32]. Furthermore, NFATc1 regulates the expression of osteoclast-specific genes such as TRAP, MMP-9, CTK, ATP6v0d2, and OSCAR [25]. MMP-9 and CTK are involved in the process of osteoclast differentiation and play an important role in osteoclast precursors and bone resorption [6, 33, 34]. MMP-9 has a negative correlation with BMD, and overexpression of MMP-9 attenuates osteoclast formation [35, 36]. CTK is a cysteine proteinase mainly expressed in osteoclasts. CTK is known to play an important role in breaking down the organic phases of bone during bone resorption [37]. According to previous studies, a deficiency of CTK indicates an osteoporosis phenotype [38]. Therefore, it was found that the deficiency of CTK is associated with the inhibition of osteoclast activity, and CTK is an effective target in the treatment of osteoporosis. ATP6v0d2 is an essential factor required for cell-cell fusion. Previous studies found ATP6v0d2-deficient mice present with an osteopetrosis phenotype due to abnormal osteoclast maturation [39, 40]. OSCAR regulates osteoclast differentiation and cell maturation and is a costimulatory receptor for osteoclast differentiation through activation of NFATc1. It is known that OSCAR may contribute to the etiology and severity of osteoporosis and rheumatoid arthritis [41, 42]. The present study showed that CPB significantly decreased the expression of osteoclast-related genes (*Nfatc1*, *Ca2*, *Acp5*, *mmp9*, *CtsK*, *Oscar*, and *Atp6v0d2*) in RANKL-induced osteoclast differentiation in RAW 264.7 cellsvia regulation of c-Fos and NFATc1 signaling.

OVX-induction is widely used in postmenopausal osteoporosis research. According to a previous study, OVX-induced rats share similar symptoms to human osteoporosis, such as the increase in body weight [43]. In addition, loss of uterus weight demonstrates that the postmenopausal osteoporosis model had been successfully established [44, 45]. In this study, all OVX-induced rats, including CPB and E_2_ treatment, increased body weight from week 4, while all groups, except for the E_2_-treatment, had decreased uterus weight. These results confirm previous studies that E_2_ treatment reverses the effect of postmenopausal changes to uterus weight, while CPB had no effect in this regard.

Micro-CT is used to analyze the structural properties of bones in three dimensions [46, 47]. Bone density and bone microstructure are indicators used to evaluate bone quality [48]. BV/TV represents the volume of bone within the volume of interest (VOI), whereas SMI refers to the structural morphology index of the cancellous bone [48, 49]. According to a recent study, increased BMD is not sufficient to improve or prevent osteoporosis [50]. Therefore, SMI is a complimentary representative index used for accurate bone quality assessment. In this study, the reduction in BMD, BV/TV, and SMI was improved by the administration of E_2_ and CPB-H. These results suggest that CPB can be a treatment for postmenopausal osteoporosis through the prevention of bone loss.

As a result of histological examination, we showed that E_2_ and CPB treatment prevented the decrease in the trabecular area, indicating that CPB inhibits bone loss of postmenopausal osteoporosis. IHC staining was used to measure the expression of bone-related factors in tissues. In this study, E_2_, CPB-L, and CPB-H groups suppressed the expression of CTK induced by OVX. Furthermore, this also correlates with the findings of the *in vitro* experiments. These results further suggest that CPB inhibits bone resorption. In summary, CPB has antiosteoporotic effects on OVX-induced rats by suppressing BMD and bone resorption markers such as CTK.

The limitations of this study are as follows: (i) MAPK and NF-*κ*B signaling pathways play an important role in NFATc1 and c-Fos activation. In this study, CPB significantly inhibited the expression of NFATc1 and c-Fos, but MAPK and NF-*κ*B pathways were not studied. Therefore, it is still necessary to correlate the inhibitory effect of CPB to the MAPK and NF-*κ*B signaling pathways. (ii) As patients with osteoporosis have already lost a certain amount of bone density, it is important to also promote osteoblast activity to restore the lost bone mass, along with osteoclast activity inhibitors to prevent disease progression. Therefore, future studies should focus on the effects of CPB on promoting osteoblast differentiation. (iii) Treatment of osteoporosis remains focused on postmenopausal osteoporosis in type 1 osteoporosis. However, interest in male osteoporosis and senile osteoporosis is also increasing. Therefore, studies on CPB in other osteoporosis models are also required.

## 5. Conclusion

In this study, CPB effectively inhibited osteoclast differentiation *in vitro* and prevented bone loss *in vivo*. The mechanisms of inhibition were via suppression of osteoporosis-related protein expression (NFATc1 and c-Fos), gene expression (*Nfatc1*, *Ca2*, *Acp5*, *mmp9*, *CtsK*, *Oscar*, and *Atp6v0d2*), and inhibited bone loss induced in the OVX model. These results indicate that CPB may be useful in the treatment of metabolic bone diseases such as osteoporosis.

## Figures and Tables

**Table 1:** Primer sequences for RT-qPCR.

Gene name	Primer sequence (5′-3′)	Tm (°C)	cycle	Accession no
MMP-9 (*Mmp9*)	F: CGA CTT TTG TGG TCT TCC CC	58	30	NM_013599.4
	R: TGA AGG TTT GGA ATC GAC CC			
CTK (*Ctsk*)	F: AGG CGG CTA TAT GAC CAC TG	58	26	NM_007802.4
	R: CCG AGC CAA GAG AGC ATA TC			
TRAP (*Acp5*)	F: ACT TCC CCA GCC CTT ACT ACC G	58	30	NM_007388.3
	R: TCA GCA CAT AGC CCA CAC CG			
NFATc1 (*Nfatc1*)	F: TGC TCC TCC TCC TGC TGC TC	58	32	NM_198429.2
	R: CGT CTT CCA CCT CCA CGT CG			
OSCAR (*Oscar*)	F: CTG CTG GTA ACG GAT CAG CTC CCC AGA	53	35	NM_001290377.1
	R: CCA AGG AGC CAG AAC CTT CGA AAC T			
CA2 (*Ca2*)	F: CTC TCA GGA CAA TGC AGT GCT GA	58	32	NM_001357334.1
	R: ATC CAG GTC ACA CAT TCC AGC A			
ATP6v0d2 (*Atp6v0d2*)	F: ATG GGG CCT TGC AAA AGA AAT CTG	58	30	NM_175406.3
	R: CGA CAG CGT CAA ACA AAG GCT TGT A			
GAPDH (*Gapdh*)	F: ACT TTG TCA AGC TCA TTT CC	58	30	NM_008084.3
	R: TGC AGC GAA CTT TAT TGA TG			

## Data Availability

All data generated or analyzed during this study are included in this published article.

## Abbreviations

SERM: Selective estrogen receptor modulators
CPB: *Crataegus pinnatifida* Bunge
OVX: Ovariectomized
Micro-CT: Microcomputed tomography
RANKL: Receptor activator of nuclear factor Kappa-B ligand
TRAF6: TNF receptor-associated factor 6
MAPK: Mitogen-activated protein kinase
NFATc1: Nuclear factor-activated T cells c1
DMEM: Dulbecco's modified eagle's medium
*α*-MEM: Minimum essential medium eagle alpha-modification
DPBS: Dulbecco's phosphate buffered saline
FBS: Fetal bovine serum
BCA: Bicinchoninic acid
E_2_: 17*β*-estradiol
MTS: Cell titer 96® aqueous nonradioactive cell proliferation assay
ABC: Avidin-biotin complex
DAB: 3, 3′-diaminobenzidine
DW: Distilled water
HPLC: High-performance liquid chromatography
SD-rat: Sprague–Dawley rat
BMD: Bone mineral density
BV/TV: bone volume/total volume
SMI: Structure model index
H&E: Hematoxylin and eosin
EDTA: Ethylenediaminetetraacetic acid
IHC: Immunohistochemistry.
